# Gorilla optimization algorithm combining sine cosine and cauchy variations and its engineering applications

**DOI:** 10.1038/s41598-024-58431-x

**Published:** 2024-03-30

**Authors:** Shuxin Wang, Li Cao, Yaodan Chen, Changzu Chen, Yinggao Yue, Wenwei Zhu

**Affiliations:** 1 School of Intelligent Manufacturing, Shanghai Zhongqiao Vocational and Technical University, Shanghai, 201514 China; 2 School of Intelligent Manufacturing and Electronic Engineering, Wenzhou University of Technology, Wenzhou, 325035 China; 3 Wenzhou Key Laboratory of New Energy Materials and Devices, Wenzhou University of Technology, Wenzhou, 325035 China

**Keywords:** Artificial gorilla troops optimizer, Refraction reverse learning, Sine and cosine algorithms, Cauchy mutation, Engineering design problems, Computational biology and bioinformatics, Evolution, Energy science and technology, Engineering, Mathematics and computing

## Abstract

To address the issues of lacking ability, loss of population diversity, and tendency to fall into the local extreme value in the later stage of optimization searching, resulting in slow convergence and lack of exploration ability of the artificial gorilla troops optimizer algorithm (AGTO), this paper proposes a gorilla search algorithm that integrates the positive cosine and Cauchy's variance (SCAGTO). Firstly, the population is initialized using the refractive reverse learning mechanism to increase species diversity. A positive cosine strategy and nonlinearly decreasing search and weight factors are introduced into the finder position update to coordinate the global and local optimization ability of the algorithm. The follower position is updated by introducing Cauchy variation to perturb the optimal solution, thereby improving the algorithm's ability to obtain the global optimal solution. The SCAGTO algorithm is evaluated using 30 classical test functions of Test Functions 2018 in terms of convergence speed, convergence accuracy, average absolute error, and other indexes, and two engineering design optimization problems, namely, the pressure vessel optimization design problem and the welded beam design problem, are introduced for verification. The experimental results demonstrate that the improved gorilla search algorithm significantly enhances convergence speed and optimization accuracy, and exhibits good robustness. The SCAGTO algorithm demonstrates certain solution advantages in optimizing the pressure vessel design problem and welded beam design problem, verifying the superior optimization ability and engineering practicality of the SCAGTO algorithm.

## Introduction

In scientific and engineering design, there are numerous complex optimization problems that are non-convex, highly nonlinear, multi-peaked, and multi-variable. Intelligent optimization algorithms have the advantages of simple programming, flexible operation, and high efficiency in optimization searches^1^. They have become a research hotspot in handling various complex optimization problems in engineering applications and have been successfully applied to solve practical problems such as neural networks^2^, resource allocation^3^, and target tracking^4^. In production life, nonlinear, high-dimensional, and irreducible multi-objective complex optimization problems often occur. These optimization problems have conflicting optimization objectives but need to be optimized simultaneously, and are therefore called multi-objective optimization problems^5,6^. In engineering practice, it is difficult to obtain optimal solutions for multiple objectives due to multiple factors. To better obtain the optimal solution set for multi-objective optimization, multi-objective evolutionary algorithms that can obtain multiple solutions after one learning process have been widely researched and applied in recent years^7–9^. Swarm intelligence optimization algorithms have the advantages of few parameters, simple implementation, and no gradient information. By simulating the behaviors of plants and animals in nature, such as courtship and foraging, the optimal solutions of these problems can be found well within a reasonable time and under highly complex constraints^10–12^. Classical swarm intelligence algorithms include particle swarm optimization (PSO)^13^, artificial bee colony (ABC)^14^, grey wolf optimization (GWO)^15^, harris hawk algorithm (HHO)^16^, and whale optimization algorithm (WOA)^17^. With the continuous development and update of technology, swarm intelligence algorithms have excelled in problems such as positioning computation^18^, path planning for travelers^19^, support vector machine optimization^20^, robotic route finding^21^, power system control^22^, and optimization of routing protocols for the Internet of Things (IoT)^23^.

Artificial Gorilla Troops Optimizer (AGTO) was proposed in 2021 by Abdollahzadeh et al.^24^. It simulates gorilla foraging and mate competition in nature^25^. Though having advantages like simple principle, easy implementation, and few adjustment parameters, it also has drawbacks like prone to local optima and slow convergence. Since its proposal, many scholars have made improvements^26^. Compared to most intelligent optimization algorithms, AGTO has certain advantages in optimization, but still has problems like low convergence accuracy and difficulty in escaping local extremes. For example, El Houd et al.^27^ proposed a mirror-opposite and adaptive mountain climbing-based gorilla optimization algorithm, using convex lens imaging reverse learning to expand the search range and avoiding local optima. Meanwhile, an adaptive mountain climbing algorithm is combined with AGTO to improve solution accuracy. Xiao et al. proposed a reverse-learning and parallel-strategy-based artificial gorilla optimization algorithm, expanding the exploration space through reverse learning and improving the global search ability^28^. The parallel strategy divides the population into multiple groups for exploration, increasing population diversity. Wu et al. utilized quadratic interpolation-based longhorn whisker search to enhance position diversity of silver-backed gorillas. They introduced a teaching and optimization algorithm with a 50% probability to update behavior following silver-backed gorillas. Finally, quasi-reflective learning generates the quasi-reflective position of silver-backed gorillas^29^. Wang et al. proposed an enhanced gorilla, using circle chaotic mapping to increase gorilla population diversity and applying it to clustering protocols in unmanned aerial vehicle-assisted intelligent vehicle networks^30^. Mostafa et al.^31^ introduced elite reverse learning to enhance population diversity, using the fusion of Cauchy inverse cumulative distribution operator and tangent flight to improve population development ability, thereby increasing convergence speed.

The motivation of this study is that, despite the aforementioned research having enhanced the optimization accuracy and speed of the gorilla algorithm, there are still shortcomings due to the short proposal time and incomplete improvement methods^32^. (1) Initial population before algorithm iteration update relies heavily on initial conditions, resulting in insufficient robustness. (2) Iterating and updating individual populations' strategies are relatively single and mechanical, failing to achieve targeted treatment and lacking a reasonable balance between global and local search; (3) The algorithm is still prone to local optimal traps, resulting in low convergence accuracy; (4) Diversity of evaluation indicators for individual populations is poor, relying solely on the fitness function to reject individual populations; (5) Individual population position update methods are not detailed enough. Therefore, further research is needed to improve the gorilla algorithm^33^.

The contributions of this study can be summarized as follows: 1. In the realm of intelligent optimization algorithms, enhancing population diversity, harmonizing global and local search, and optimizing the process of updating individual population positions are all effective approaches to enhance algorithm optimization performance. 2. This study presents an AGTO based on Sine Cosine and Cauchy Mutation (SCAGTO), which integrates sine cosine and Cauchy mutations. Firstly, refracted reverse learning is employed to generate the initial population, thereby increasing the diversity of the initial population. Secondly, in the algorithm optimization stage, the Sine Cosine Algorithm (SCA) is introduced to update the position of the discoverer. By leveraging the oscillation characteristics of the sine cosine model to influence the position of the discoverer, the diversity of the discoverer is maintained, consequently enhancing the global search ability and convergence speed of the AGTO. 3. Finally, Cauchy mutation is utilized to perturb individuals in the gorilla position updates, expanding the search scale of the gorilla algorithm and improving the algorithm's capacity to escape local optima and update individual positions, thereby enhancing convergence accuracy. The superiority of the algorithm is verified through the analysis of the benchmark test function. Finally, via the analysis of experimental results on engineering application problems, the SCAGTO algorithm is demonstrated to be more feasible compared to other algorithms.

## Artificial gorilla troops optimizer algorithm

**Figure 1:**
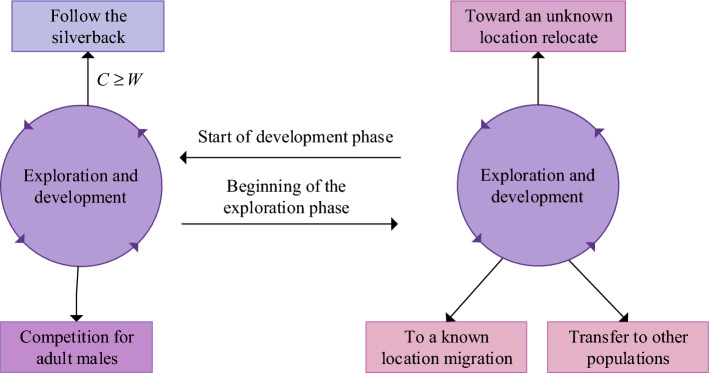
Exploration and Development Mechanism of AGTO.

The artificial gorilla troop optimization algorithm (AGTO) is a population intelligence optimization algorithm proposed based on the collective lifestyle and social behavior of gorillas^34^. Gorilla live in a group called "troops", consisting of a group of adult male or silver backed guerillas and several adult female gorillas and their descendants. The silver backed gorilla is the core of the group, making all decisions, mediating battles, determining group actions, guiding the gorilla in finding food sources, and taking responsibility for the safety and well-being of the group^35,36^. In the AGTO algorithm, five different operators are used to simulate the collective behavior of gorillas, mainly divided into two stages: exploration stage and development stage. The exploration phase employed three different mechanisms, namely migration to unknown locations, migration to other gorilla locations, and migration to known locations^37^. During the development phase, two social behaviors were adopted: following the silver backed gorilla and competing with adult female gorillas. Figure 1 shows the exploration and development mechanism of optimization algorithms for artificial gorilla troops.

The position update of gorillas during the exploration phase is shown in Eqs. (1), (2), (3):

1 $$ GX(t + 1) = (ub - lb) \times r_{1} + lb, \, rand < s \, $$

2 $$ GX(t + 1) = (r_{2} - C) \times X_{r} (t) + L \times H, \, rand \ge 0.5 $$

3 $$ GX(t + 1) = X(t) - L \times (L \times X(t) - GX_{r} (t)) + r_{3} \times (X(t) - GX_{r} (t))), \, rand < 0.5 $$

In the formula, *GX*(*t* + 1) is the candidate position for the gorilla in the next iteration, and *X*(*t*) is the current position of the gorilla. In addition, *r*1, *r*2, *r*3, and *rand* are random values with values of (0,1). *s* is a parameter that must be given a value before optimizing operations, usually with a value of 0.03. This parameter determines the probability of the gorilla migrating to an unknown location^38^. *ub* and *lb* represent the upper and lower limits of variables, respectively. *Xr* and *GXr* are individual positions of a gorilla randomly selected from a population. The parameters *C*, *L*, and *H* are represented by formulas (4), (5), and (6) respectively:

4 $$ C = \left( {\cos (2 \times r_{4} ) + 1} \right) \times \left( {1 - \frac{It}{{MaxIt}}} \right) $$

5 $$ L = C \times l $$

6 $$ H = Z \times X(t) $$

In Eq. (4), the parameter of *It* is the current iteration value, and *MaxIt* is the total iteration value for executing the optimization operation. *r*_4_ is a random value with a value of (0,1). In Eq. (5), the parameter of *l* is a random value with a value of (− 1, 1). In Eq. (6), the parameter of *Z* is a random value with a value of [− *C*,* C*] in the problem dimension. During the development phase, gorillas choose to follow the silver backed gorilla mechanism, and their behavior is shown in Eq. (7):

7 $$ GX(t + 1) = L \times M \times \left( {X(t) - X_{silverback} } \right) + X(t) $$

8 $$ M = \left( {\left| {\frac{1}{N} \times \sum\limits_{i = 1}^{N} {GX_{i} (t)} } \right|^{g} } \right)^{\frac{1}{g}} $$

9 $$ g = 2^{L} $$

In Eq. (7), *Xsilverback* is the position of the silver backed gorilla (best solution), and the parameter of *L* is a parameter that must be given a value before optimization operation, used to switch the development stage mechanism. The parameter of *M* is represented by Eq. (8), where *GXi*(*t*) represents the position of the *i*-th candidate gorilla at the *t*-th iteration, the parameter of *N* represents the total number of gorillas, and *g* is represented by Eq. (9). During the development phase, gorillas choose to compete with adult female gorilla mechanisms, and their behavior is shown in Eq. (10):

10 $$ GX(t + 1) = X_{silverback} - \left( {X_{silverback} \times Q - X(t) \times Q} \right) \times A $$

11 $$ Q = 2 \times r_{5} - 1 $$

12 $$ A = \beta \times E $$

13 $$ E = \left\{ {\begin{array}{*{20}c} {N_{1} ,} & {rand \ge 0.5} \\ {N_{2} , \, } & {rand < 0.5} \\ \end{array} } \right. $$

**Figure 2:**
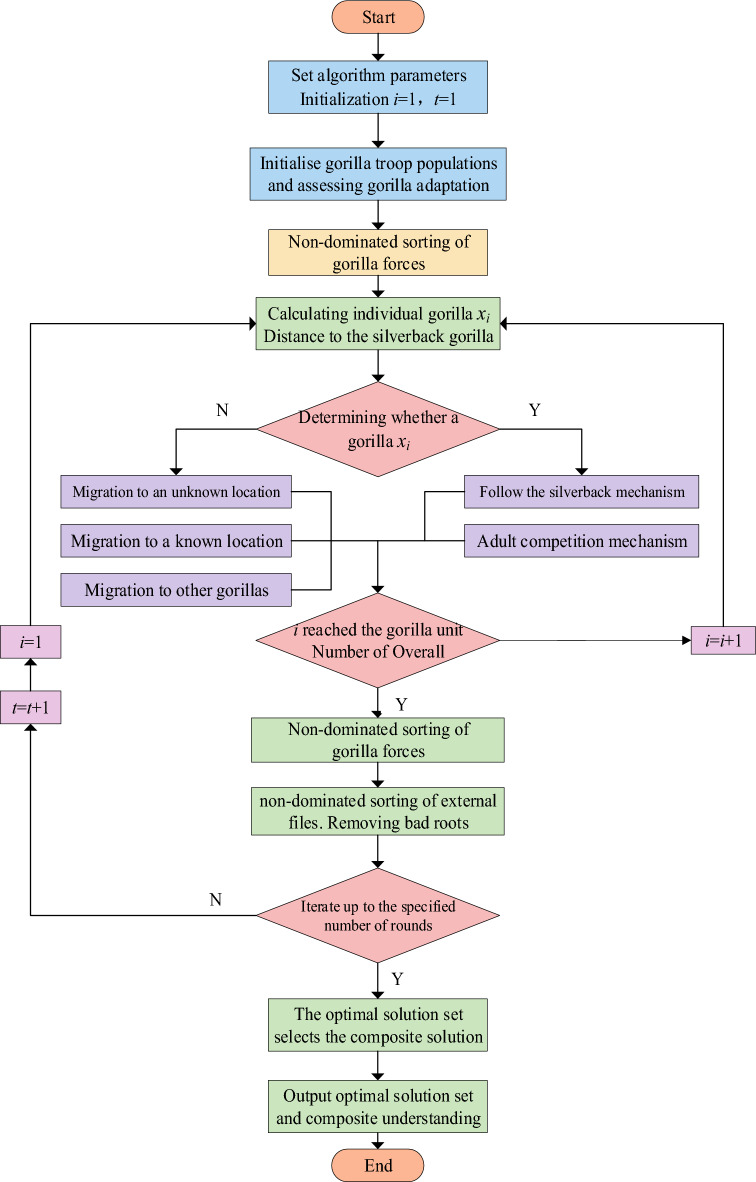
Optimization flowchart of the AGTO algorithm.

In Eq. (10), the parameter of *Q* is used to simulate the impact force, represented by Eq. (11), the parameter of *A* is the coefficient of the degree of violence in the conflict, represented by Eq. (12). In Eq. (11), *r*_5_ is a random value with a value of (0,1); In Eq. (12), $\beta$ is the parameter value given before the optimization operation. The parameter of *E* is used to simulate the impact of violence on the dimensions of the solution, represented by Eq. (13). If *rand* is 0.5, the parameter of *E* will be equal to the normal distribution and the random value in the problem dimension. Conversely, the parameter of *E* will be equal to the random value in the normal distribution. When improving the multi-objective gorilla troop optimization algorithm in this article, the calculation of gorilla troop coding, population initialization, coding factor, and crowding distance is shown in Fig. 2. Figure 2 shows the optimization algorithm flowchart for the gorilla unit.

## AGTO algorithm integrates sine and cosine and Cauchy mutation

### Refractive reverse learning strategy

**Figure 3:**
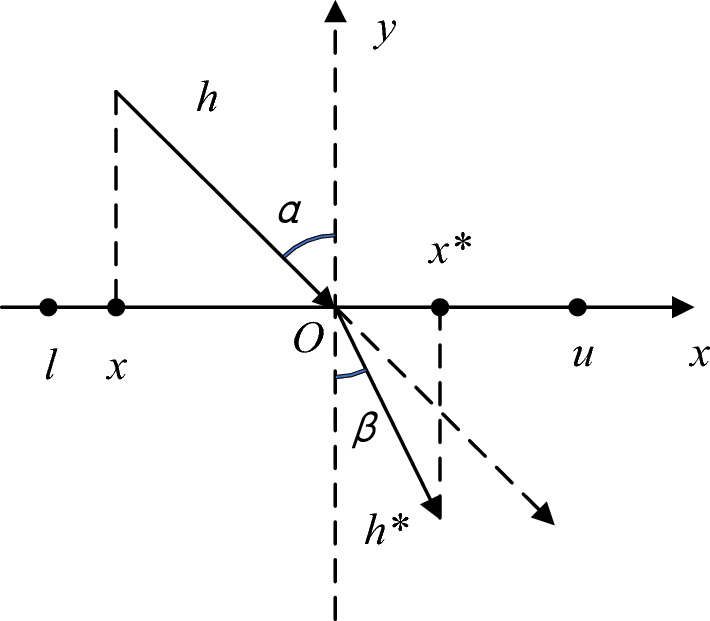
Refractive reverse learning principle.

In view of the loss of population diversity of AGTO algorithm in the late stage of optimization, which increases the probability of falling into local extremum and leads to the problem of insufficient convergence accuracy, this paper uses a refraction reverse learning mechanism to initialize the gorilla force algorithm population^39^. Reverse learning is an optimization strategy proposed by tizhoosh. The basic idea is to expand the search scope by calculating the reverse solution of the current solution, so as to find a better alternative solution for a given problem. The combination of intelligent algorithm and reverse learning can effectively improve the accuracy of the algorithm^40^. At the same time, reverse learning still has some shortcomings. The introduction of reverse learning in the early stage of optimization can strengthen the convergence performance of the algorithm, but it is easy to make the algorithm fall into premature convergence in the later stage. Therefore, a refraction principle is introduced into the reverse learning strategy to reduce the probability of premature convergence in the later stage of the search. The principle of refraction reverse learning is shown in Fig. 3.

Where, the optimisation range of the solution above the *x*-axis is [*l*, *u*], the *y*-axis is the normal line, *α* and *β* denote the angles of incidence and refraction, *h* and h^*^ are the lengths of the incident and refracted light rays respectively, and the parameter *O* is the midpoint of the optimisation range [*l*, *u*]. According to the geometric relationship of lines in mathematics, the following is obtained:

14 $$ \left\{ \begin{gathered} \sin \alpha = \left( {(l + u)/2} \right) - x)/h \hfill \\ \sin \beta = \left( {x^{*} - (l + u)/2} \right)/h^{*} \hfill \\ \end{gathered} \right. $$

According to the definition of refractive index, $n = \sin \alpha /\sin \beta$, the formula for refractive index *n* is obtained as:

15 $$ n = \frac{{h^{*} (l + u)/2) - x)}}{{h(x^{*} - (l + u)/2)}} $$

Let the scaling factor $k = h/h^{*}$ be substituted into (15) to obtain the deformation equation as

16 $$ x^{*} = \frac{l + u}{2} + \frac{l + u}{{2kn}} - \frac{x}{kn} $$

When *n* = 1 and *k* = 1, Eq. (16) can be converted to a reverse learning formula:

17 $$ x^{*} = l + u - x $$

Equation (16) when generalized to the high dimensional space of the Gorilla Force algorithm, making *n* = 1 gives

18 $$ x^{*}_{i,j} = \frac{{l_{j} + u_{j} }}{2} + \frac{{l_{j} + u_{j} }}{2kn} - \frac{{x_{i,j} }}{kn} $$

In Eq. (18), the parameter $x_{i,j}$ is the *i*-th gorilla in the population in the *j*-th dimensional position (*i* = 1, 2, 3,$\cdots$,*D*,* j* = 1, 2, 3,$\cdots$,*D*), the parameter* D* is the number of populations, the spatial dimensionality of the *N* solution, and $x^{*}_{i,j}$ is the $x_{i,j}$ refractive direction position. the parameters *l*_*j*_ and *u*_*j*_ are the minimum and maximum values in the *j*-th dimension of the search space respectively.

### Sine–cosine strategy

In the process of gorilla predation, the location of food source plays a very important role, affecting the direction of the whole gorilla population. However, considering that the food sources may be different and the locations may be different, when the food that the discoverer is searching for is at the local optimum, a large number of followers will pour into the location. At this time, the discoverer and the whole population stagnate, resulting in the loss of population position diversity, and then increasing the possibility of falling into local extremum. In view of this phenomenon, this paper introduces sine cosine algorithm (SCA) in the location update of the discoverer of gorilla search algorithm^41^, which can maintain the individual diversity of the discoverer and improve the global search ability of agto by using the oscillation characteristics of the sine cosine model. The central idea of SCA is to seek global and local optimizations according to the oscillation changes of the sine and cosine model to obtain the global optimal value^42^.

**Figure 4:**
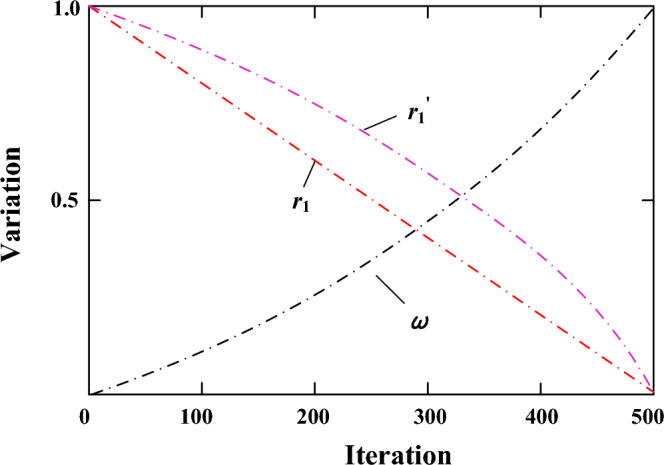
Variation curves of *r*_*1*_, *r*_*1*_',* ω*.

For the step size search factor *r*_1_ = *a -at*/*Iter*max (*a* is a constant, *t* is the number of iterations, and this paper sets *a* = 1) of the basic sine and cosine algorithm shows a linear decreasing trend, which is not conducive to further balancing the global search and local development capabilities of AGTO algorithm. Inspired by literature^43^, the step size search factor is improved, and the transformation curve is shown in Fig. 4. The new nonlinear decreasing search factor is shown in Eq. (19), which has a large weight in the early stage and a slow decreasing speed, It is beneficial to improve the global optimization ability. When the weight factor is small, it enhances the advantages of the algorithm in local development and speeds up the speed of obtaining the optimal solution.

19 $$ r^{\prime}_{1} = a \times \left( {1 - \left( {\frac{t}{{Iter_{\max } }}} \right)^{\eta } } \right)^{{\frac{1}{\eta }}} $$

wherein, $\eta$ is the adjustment coefficient,$\eta$ $\ge$ 1, *a* = 1.

In the whole search process of the AGTO algorithm, the update of individual position of population is often affected by the current position. Therefore, the nonlinear weighting factor *ω* of Eq. (20) is introduced. It is used to adjust the dependence of the individual position update of the population on the individual information currently. In the early stage of optimization, the smaller ω It reduces the influence of individual location update on the current solution location, and improves the global optimization ability of the algorithm. In the later stage, the larger ω Taking advantage of the high dependence of current location information and individual location update, the convergence speed of the algorithm is accelerated, and the change curve is shown in Fig. 4. Then a new finder location update formula is obtained, as shown in formula (21):

20 $$ \omega = \frac{{e^{{\frac{t}{{Iter_{\max } }}}} - 1}}{e - 1} $$

21 $$ X_{i,j}^{t + 1} = \left\{ \begin{gathered} \omega \cdot X_{i,j}^{t} + r^{\prime}_{1} \cdot \sin r_{2} \left| {r_{3} \cdot X_{best} - X_{i,j}^{t} } \right|,R_{2} < ST \hfill \\ \omega \cdot X_{i,j}^{t} + r^{\prime}_{1} \cdot \cos r_{2} \left| {r_{3} \cdot X_{best} - X_{i,j}^{t} } \right|,R_{2} \ge ST \hfill \\ \end{gathered} \right. $$

wherein, a random number of *r*_2_ ∈ [0, 2π] determines the distance moved by the gorilla, and a random number of *r*_3_ ∈ [0, 2π] controls the effect of the optimal individual on the latter position of the gorilla.

### Cauchy variation strategy

In the process of foraging, followers often forage around the best discoverer, and there may also be food competition, making themselves discoverers^44^. To avoid the algorithm falling into local optimization, Cauchy mutation strategy is introduced into the follower update formula to improve the global optimization ability. The new follower position is updated as follows:

22 $$ X_{i,j}^{t + 1} = X_{best} (t) + cauchy(0,1) \oplus X_{best} (t) $$

where $cauchy(0,1)$ is the standard Cauchy distribution function and $\oplus$ denotes the multiplicative implication.

The one-dimensional Cauchy variation function centered at the origin is as follows:

23 $$ f(x) = \frac{1}{\pi }\left( {\frac{1}{{x^{2} + 1}}} \right), - \infty < x < \infty $$

The Cauchy distribution is similar to the standard normal distribution, which is a continuous probability distribution with small values at the origin, relatively flat and long at both ends, and the rate of approaching zero is slow, so it can produce greater disturbance than the normal distribution^45^. Therefore, Cauchy mutation is used to perturb the individuals in the gorilla position update, so as to expand the search scale of gorilla algorithm, and then improve the ability of the algorithm to jump out of the local optimum.

### Implementation steps of the SCAGTO algorithm

The development method of the gorilla force involves selecting several vectors of the silverback gorilla for position optimization to achieve the optimization of position vectors close to itself. The detailed steps of the optimization improvement mechanism are as follows:

1. Initialize relevant parameters: Set the population size to *N*, the dimension to dim, the maximum number of iterations to *MaxIt*, the population search range to *u*_*b*_ and *l*_*b*_, the exploration phase switching probability to *s*, the development phase switching probability to *w*, and the parameter *β*.
2. Initialize the population *X* using the refraction reverse learning strategy.
3. Calculate the fitness value of each gorilla individual *X*, and select the individual with the smallest fitness as the silverback gorilla *Xsilverback*.
4. Calculate the values of parameters *C* and *L* according to formulas (4) and (5).
5. Update the gorilla candidate location *GX* based on formulas (1), (2), (10), and the switching probability s in the exploration phase, and check the boundary of the updated candidate location *GX*. According to the gorilla search algorithm, the sine and cosine algorithm is introduced into the location update of the discoverer. By utilizing the oscillation characteristics of the sine and cosine model, the location of the discoverer is influenced to maintain the individual diversity of the discoverer, thereby enhancing the global search ability of the AGTO algorithm. Update the position of the gorilla *X*, and select the gorilla with the least fitness as the silverback gorilla *Xsilverback*.
6. Update the gorilla candidate location *GX* according to formulas (7) and (10) and the development phase switching probability w, check the boundary of the updated location *GX*, update the gorilla location x using the dimension-by-dimension update strategy, and select the gorilla with smaller fitness as the silverback gorilla *Xsilverback*.
7. Apply the Cauchy mutation strategy to the individual that may fall into the local optimum according to formulas (22) and (23), enhance the global optimization ability, update the position of the gorilla again based on the dimension-by-dimension update strategy, and select the gorilla with smaller fitness as the silverback gorilla *Xsilverback*.
8. Determine whether the iteration termination conditions are met. If so, output the silverback gorilla position *Xsilverback* and its fitness value. Otherwise, proceed to step 4 to continue.

### Complexity analysis of the SCAGTO algorithm

The time complexity indirectly reflects the convergence speed of the algorithm. In the gorilla force algorithm, the time complexity of the initialization process (population size n, maximum number of iterations T, search space dimension *D* is *O*(*N*), and all solutions are updated to the optimal solution in the exploration and development phase. The time complexity of this process is *O*(*T* × *N*) + *O*(*T* × *N* × *D*) × 2. Then the time complexity of the standard artificial gorilla force is:

24 $$ O\left( {{\text{AGTO}}} \right) \, = O\left( {T \times D} \right) \, + O\left( N \right) \, + O\left( {T \times N \times D} \right) \, \times {2 } = O\left( {N \times D} \right) $$

In the improved gorilla force algorithm, the initialization parameters are consistent with the standard artificial gorilla force algorithm. If the external file set is used to store the non-dominated solution, the time complexity of the initialization process is *O*(*N*).

In the stage of algorithm exploration and development, the SCAGTO algorithm introduces refraction reverse learning strategy to replace random initialization with *O*(*N* × *D*). The individual fitness is the same as the AGTO algorithm, the introduction of sine and cosine phase requires *O*(*N* × *D*), and the complexity of Cauchy variation phase is *O*(*N* × *D*). Therefore, the total complexity of the improved SCAGTO algorithm is:

25 $$ O\left( {{\text{SCAGTO}}} \right) \, = O\left( {T \times D} \right) \, + O\left( N \right) \, + O\left( {T \times N \times D} \right) \, \times {2 } = O\left( {N \times D} \right) $$

The improved artificial gorilla force algorithm has the same time complexity as the standard artificial gorilla force algorithm. The improved strategy proposed in this paper does not increase the time complexity of the standard artificial gorilla force algorithm.

## Simulation comparison and performance test

### Test function experiment comparison

To validate the performance of the proposed SCAGTO algorithm, which combines sine and cosine with Cauchy mutations, a detailed comparison was conducted with six algorithms, including the whale optimization algorithm (WOA)^46^, harris hawks optimization (HHO)^47^, grey wolf optimization algorithm (GWO)^48^, ant lion colony optimization algorithm (ALO)^49^, African vultures optimization algorithm (AVOA)^50^, and the basic Artificial gorilla troops optimizer (AGTO)^24^. A comprehensive comparison was carried out under 30 classic test functions in the CEC2018 test suite presented in Table 1. The CEC2018 encompasses a total of 14 test functions: DF1-DF14, with DF1-DF9 being two-objective and DF10-DF14 being three-objective^51^. Table 2 presents the primary parameter configurations for the seven algorithms. The experimental environment utilized is Windows 10, a 64-bit operating system, with the processor being an Intel^®^ Core™ i7-10870H CPU operating at a frequency of 2.75 GHz. The algorithm is based on MATLAB 2022b and implemented in the M language. The CEC2018 test suite consists of a total of 30 single-objective test functions, with search intervals ranging between [− 100, 100]^52^. All the test functions aim to solve the minimization problem, with D representing the dimension (30 dimensions), as follows:

**Table 1:** Test functions.

Problem	Objectives	Dynamics	Remarks
DF1	2	Mixed convexity-concavity, location of optima	Dynamic PF and PS
DF2	2	Switch of position-related variable, location of optima	Static convex PF, dynamic PS, severe diversity loss
DF3	2	Mixed convexity-concavity, variable-linkage, location of optima	Dynamic PF and Ps
DF4	2	Variable-linkage, PF range, bounds of PS	Dynamic PF and Ps
DF5	2	Number of knee regions, local of optima	Dynamic PF and Ps
DF6	2	Mixed convexity-concavity, multimodality, location of optima	Dynamic PF and Ps
DF7	2	PF range, location of optima	Convex PF, static PS centroid, dynamic PF and PS
DF8	2	Mixed convexity-concavity, distribution of solutions, location of optima	Static PS centroid, dynamic PF and PS, variable-linkage
DF9	2	Number of disconnected PF segments, location of optima	Dynamic PS and PF, variable-linkage
DF10	3	Mixed convexity-concavity, location of optima	Dynamic PS and PE, variable-linkage
DF11	3	Size of PF region, PF range, location of optima	Dynamic PS and PF, concave PF, variable-linkage
DF12	3	Number of PF holes, location of optima	Dynamic PS, static concave PF, variable-linkage
DF13	3	Number of disconnected PF segments, location of optima	Dynamic PS and PF, the PF can be a continuous convex or concave segment, or several disconnected segments
DF14	3	Degenerate PF, number of knee regions, location of optima	Dynamic PS and PF, variable-linkage

**Table 2:** Comparison of seven algorithms for optimal value of *Min*.

Function	WOA	HHO	GWO	ALO	AVOA	AGTO	SCAGTO
F1	4.19E + 09	1.07E + 09	3.23E + 09	1.92E + 08	4.98E + 05	1.01E + 09	1.52E + 06
F2	0	0	0	0	0	0	0
F3	3.07E + 05	6.36E + 04	4.74E + 04	1.59E + 05	5.38E + 04	6.29E + 04	8.02E + 03
F4	7.56E + 02	7.79E + 02	5.48E + 02	5.29E + 02	5.36E + 02	5.90E + 02	5.68E + 02
F5	8.61E + 02	7.42E + 02	6.23E + 02	7.82E + 02	6.80E + 02	8.08E + 02	7.15E + 02
F6	6.67E + 02	6.70E + 02	6.15E + 02	6.48E + 02	6.61E + 02	6.58E + 02	6.49E + 02
F7	1.38E + 03	1.27E + 03	8.63E + 02	1.17E + 03	1.15E + 03	1.33E + 03	9.65E + 02
F8	1.04E + 03	9.88E + 02	1.05E + 03	9.38E + 02	9.53E + 02	9.60E + 02	9.42E + 02
F9	1.57E + 04	8.42E + 03	1.18E + 03	3.98E + 03	5.06E + 03	5.59E + 03	4.77E + 03
F10	6.80E + 03	6.33E + 03	4.20E + 03	5.64E + 03	5.97E + 03	7.84E + 03	5.01E + 03
F11	6.92E + 03	1.54E + 03	1.90E + 03	1.81E + 03	1.22E + 03	1.77E + 03	1.22E + 03
F12	8.62E + 08	5.04E + 07	3.94E + 06	9.20E + 06	1.00E + 07	1.71E + 07	2.43E + 06
F13	9.06E + 06	2.41E + 05	1.61E + 05	2.88E + 04	7.16E + 04	8.64E + 04	3.03E + 03
F14	3.65E + 06	1.09E + 06	9.31E + 05	8.15E + 04	4.56E + 05	2.86E + 05	1.10E + 04
F15	5.67E + 05	8.31E + 04	5.74E + 04	3.53E + 04	2.25E + 04	7.88E + 03	1.79E + 03
F16	4.33E + 03	3.19E + 03	2.57E + 03	2.87E + 03	3.45E + 03	3.26E + 03	3.24E + 03
F17	2.76E + 03	2.69E + 03	1.92E + 03	2.73E + 03	2.39E + 03	1.87E + 03	2.20E + 03
F18	9.49E + 06	6.25E + 06	6.04E + 06	2.61E + 06	2.00E + 07	1.99E + 06	2.48E + 05
F19	1.14E + 07	1.34E + 06	3.56E + 06	6.77E + 05	2.70E + 04	8.95E + 03	4.69E + 03
F20	2.67E + 03	2.94E + 03	2.46E + 03	2.97E + 03	2.48E + 03	2.86E + 03	2.46E + 03
F21	2.79E + 03	2.62E + 03	2.38E + 03	2.42E + 03	2.43E + 03	2.45E + 03	2.42E + 03
F22	1.02E + 04	7.75E + 03	5.69E + 03	2.47E + 03	5.87E + 03	2.50E + 03	2.32E + 03
F23	3.07E + 03	3.27E + 03	2.79E + 03	2.87E + 03	2.91E + 03	2.89E + 03	2.86E + 03
F24	3.13E + 03	3.56E + 03	3.01E + 03	2.99E + 03	3.20E + 03	3.06E + 03	2.93E + 03
F25	3.30E + 03	3.02E + 03	3.02E + 03	2.95E + 03	2.95E + 03	2.96E + 03	2.95E + 03
F26	9.03E + 03	8.46E + 03	5.23E + 03	5.44E + 03	3.43E + 03	5.67E + 03	2.87E + 03
F27	3.31E + 03	3.32E + 03	3.27E + 03	3.41E + 03	3.34E + 03	3.25E + 03	3.36E + 03
F28	3.82E + 03	3.47E + 03	3.38E + 03	3.29E + 03	3.33E + 03	3.31E + 03	3.33E + 03
F29	7.10E + 03	4.57E + 03	3.65E + 03	4.72E + 03	5.05E + 03	3.92E + 03	4.29E + 03
F30	1.34E + 07	5.35E + 06	2.10E + 07	1.69E + 07	7.82E + 05	2.39E + 04	4.28E + 04

**Figure 5:**
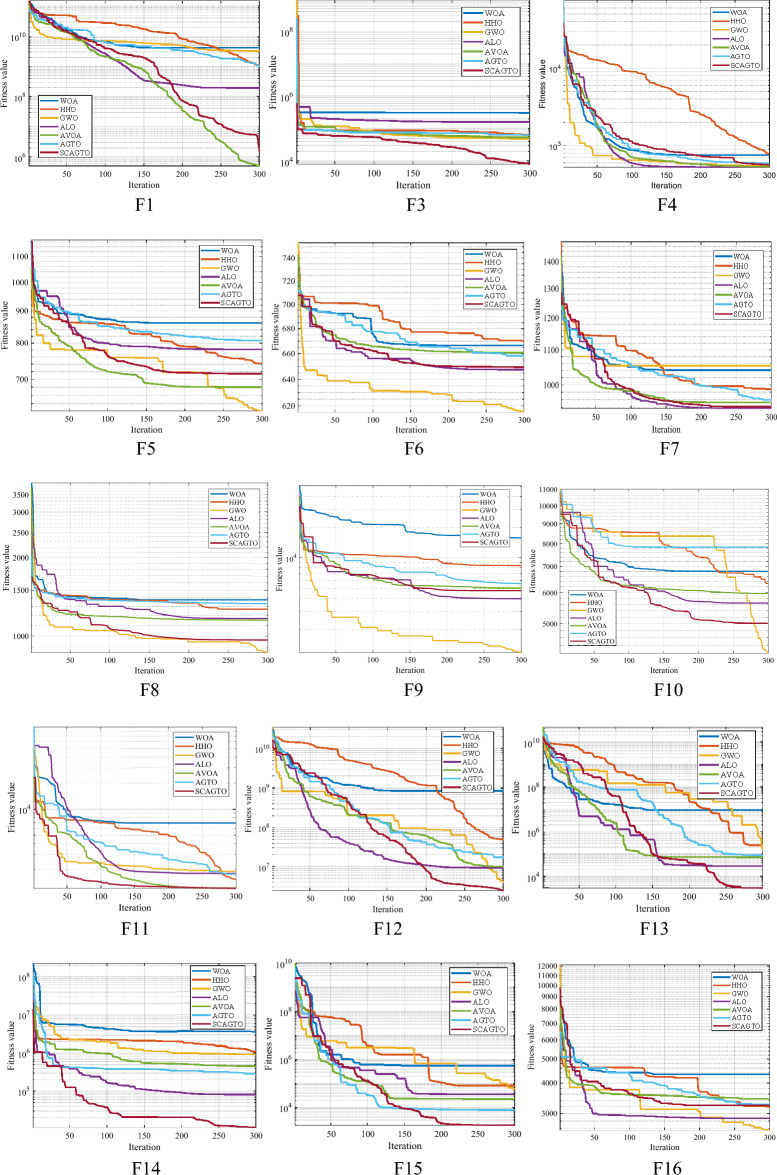

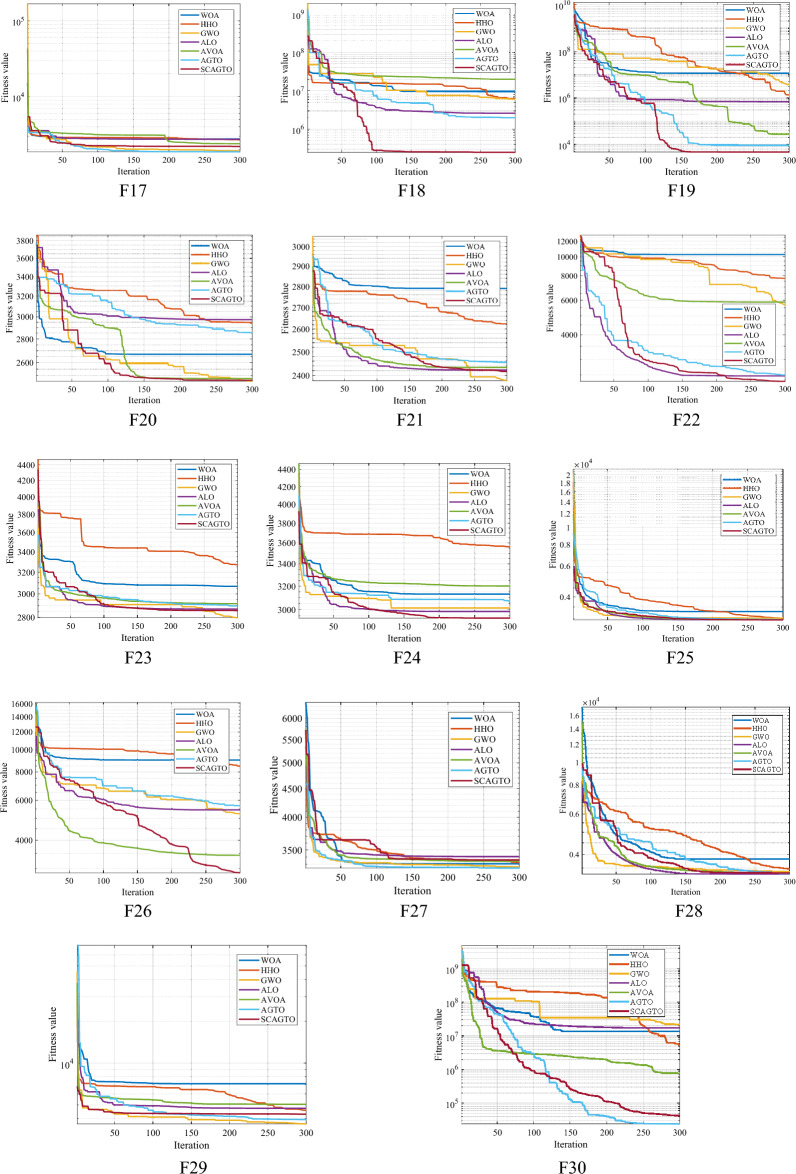
Comparison of seven iterative optimization algorithms.

In order to present a detailed account of the convergence of diverse algorithms, a convergence curve chart will be employed to accomplish this. To ensure fairness in the comparison, the population size of the seven algorithms was fixed at 30, the dimension dim was set to 30, and the maximum number of iterations was set to 500. A convergence curve of 100 independent runs was derived, and Fig. 5 illustrates the convergence curve of the 30 functions. Among them, Fig. 2 depicts the program error, and it is recognized that there is an issue with this function, thus no image of it is provided. As illustrated in Fig. 5, the base-10 logarithm is utilized as the y-axis. When the curve ceases to be displayed as the number of iterations increases, it signifies that the algorithm has attained the theoretical optimal solution of 0.

As can be seen from the single-peak convergence curve in Fig. 5, the convergence performance of AGTO is slightly better than that of WOA, HHO, GWO, ALO and AVOA algorithms, but the convergence curves also show a flat trend, stagnation, low accuracy of optimality search, and fall into the local optimum. The improved algorithm of SCAGTO has significant improvement in convergence speed and accuracy compared with that of AGTO, and it also verifies the ability of the sine–cosine strategy and the Cauchy variant to jump out of the In the overall comparison between SCAGTO and AGTO, the two are comparable in convergence speed, while SCAGTO finally shows higher convergence accuracy, mainly because of the mutation performance when searching for the optimum with the help of Cauchy's variation. The convergence speed and accuracy of SCAGTO are further improved compared to AGTO, and both obtain the theoretical optimal solution.

In terms of optimisation accuracy, AGTO algorithm has better optimisation accuracy than WOA, HHO, GWO, ALO and AVOA algorithms in different dimensions of each test function, and AGTO algorithm achieves the theoretical optimal value of 0 when solving F4, F15, F19, and F27, and AGTO achieves a better extreme value when solving F30, which is not optimal, but is still significantly better than WOA, HHO, GWO, ALO and AVOA algorithms. The SCAGTO algorithm, which is an improved strategy to AGTO, shows an advantage in solving the function when SCAGTO solves dim = 300, with the order of magnitude of the mean value improved by at least 12 and up to 30 orders of magnitude compared to AGTO. In the whole test function, the overall convergence accuracy of SCAGTO is significantly improved, and it also shows that the positive cosine strategy and the Cauchy variation strategy have a positive effect on the AGTO algorithm's ability to find the global optimum, and reduce the chance of AGTO falling into the local optimum.

In order to test the optimisation accuracy of SCAGTO, the above seven algorithms are optimized under 30 test functions (spatial dimension *dim* = 30), in which each algorithm is run independently under the function for 100 times, and the results are evaluated using six performance metrics, namely, *Min*, *Mean*, standard deviation of *Std*, *Median*, *Max*, and running time, and experimental comparative results are obtained, as shown in Tables 2, 3, 4, 5, 6, and 7.

**Table 3:** Comparison of seven algorithms for optimal value of *Mean*.

Function	WOA	HHO	GWO	ALO	AVOA	AGTO	SCAGTO
F1	− 1.44E + 00	− 4.15E + 00	2.00E + 00	− 3.47E + 00	− 6.89E − 01	8.12E − 01	8.12E − 01
F2	1.67E + 01	1.64E + 01	− 3.18E + 00	3.96E + 00	1.42E + 00	− 5.87E + 00	− 5.87E + 00
F3	− 6.10E − 01	− 8.47E + 00	4.45E + 00	7.88E + 00	− 7.86E + 00	− 2.44E + 00	− 2.44E + 00
F4	1.64E + 01	9.88E + 00	1.17E + 01	8.57E + 00	8.35E + 00	5.21E + 00	5.21E + 00
F5	1.20E + 01	9.34E + 00	− 2.38E + 00	9.20E + 00	− 1.04E + 01	− 1.99E − 01	− 1.99E − 01
F6	− 1.01E + 01	6.56E + 00	8.56E + 00	1.26E + 01	8.26E + 00	8.05E + 00	8.05E + 00
F7	− 1.94E + 00	4.01E − 01	2.25E − 02	− 1.95E + 00	1.91E + 00	− 8.13E − 01	− 8.13E − 01
F8	− 7.32E + 00	1.73E + 00	− 1.18E + 01	− 4.53E + 00	− 1.12E + 01	− 4.22E + 00	− 4.22E + 00
F9	− 9.95E + 00	− 2.36E + 00	− 5.12E + 00	9.40E + 00	2.43E + 00	6.60E + 00	6.60E + 00
F10	− 3.79E + 00	− 8.78E + 01	− 3.61E + 00	8.29E + 01	6.05E + 01	− 1.01E + 01	− 1.01E + 01
F11	− 8.53E − 01	− 1.61E + 01	− 1.05E + 01	3.32E + 00	− 8.06E + 00	− 6.26E + 00	− 6.26E + 00
F12	1.76E + 01	2.34E + 01	1.36E + 01	1.26E + 01	1.47E + 01	− 2.60E + 00	− 2.60E + 00
F13	2.14E + 01	2.68E + 01	1.36E + 01	4.11E + 01	4.85E + 01	1.94E + 00	1.94E + 00
F14	4.38E + 01	8.07E + 00	− 3.07E + 00	3.91E + 01	− 3.00E + 01	6.96E − 01	6.96E − 01
F15	9.46E + 00	1.49E + 01	3.84E + 00	− 1.77E + 00	2.25E + 01	1.05E + 01	1.05E + 01
F16	− 3.19E + 01	− 3.79E + 00	− 5.20E + 00	8.66E + 00	1.98E + 00	− 2.95E + 00	− 2.95E + 00
F17	− 1.73E + 01	− 1.51E + 01	3.22E + 00	− 1.31E + 01	− 2.33E + 01	− 4.33E + 00	− 4.33E + 00
F18	− 4.73E + 01	− 3.26E + 01	− 6.02E + 00	− 1.97E + 01	− 2.83E + 00	2.20E − 01	2.20E − 01
F19	− 4.74E + 00	1.20E + 01	6.24E + 00	4.17E + 00	8.94E + 00	3.14E − 01	3.14E − 01
F20	1.12E + 01	− 2.34E + 00	6.24E + 00	1.58E + 00	2.79E + 01	− 5.44E + 00	− 5.44E + 00
F21	1.88E + 01	2.48E + 01	2.52E + 00	9.27E + 00	1.40E + 01	3.54E + 00	3.54E + 00
F22	− 2.80E + 00	− 5.69E + 00	3.43E + 00	− 2.00E + 01	− 5.96E + 00	− 3.91E + 00	− 3.91E + 00
F23	− 2.68E + 01	− 2.20E + 01	− 1.32E + 01	− 8.50E + 00	− 3.73E + 01	− 5.09E + 00	− 5.09E + 00
F24	− 2.50E + 01	− 2.30E + 01	− 8.90E + 00	− 6.20E + 00	− 2.17E + 01	− 5.71E + 00	− 5.71E + 00
F25	1.22E + 01	1.43E + 01	1.34E + 01	1.63E + 01	1.59E + 01	1.33E + 01	1.33E + 01
F26	3.90E − 01	− 1.34E + 00	− 1.31E + 01	− 2.85E + 01	− 1.57E + 01	− 1.56E + 01	− 1.56E + 01
F27	5.26E + 00	2.48E + 01	− 3.44E − 01	9.91E − 02	9.11E + 00	− 3.54E + 00	− 3.54E + 00
F28	1.27E + 00	7.84E + 00	2.64E + 00	− 3.44E + 00	− 1.39E + 00	− 3.46E − 01	− 3.46E − 01
F29	− 1.12E + 01	1.16E + 01	8.68E + 00	3.96E + 01	− 7.04E + 00	1.68E + 00	1.68E + 00
F30	− 6.95E + 00	2.01E + 01	2.68E + 00	2.68E + 00	2.68E + 00	2.68E + 00	2.68E + 00

**Table 4:** Comparison of seven algorithms for optimal value of *Std*.

Function	WOA	HHO	GWO	ALO	AVOA	AGTO	SCAGTO
F1	3.99E + 01	4.16E + 01	4.56E + 01	4.20E + 01	4.37E + 01	4.50E + 01	4.50E + 01
F2	5.81E + 01	4.65E + 01	6.04E + 01	6.21E + 01	5.79E + 01	5.26E + 01	5.26E + 01
F3	6.02E + 01	2.28E + 01	2.55E + 01	6.47E + 01	2.49E + 01	2.74E + 01	2.74E + 01
F4	4.83E + 01	4.83E + 01	5.40E + 01	4.09E + 01	5.26E + 01	5.15E + 01	5.15E + 01
F5	4.67E + 01	3.39E + 01	3.42E + 01	3.47E + 01	4.17E + 01	4.32E + 01	4.32E + 01
F6	3.41E + 01	1.83E + 01	4.55E + 01	3.43E + 01	4.43E + 01	4.64E + 01	4.64E + 01
F7	1.00E + 01	7.43E + 00	2.14E + 01	2.26E + 01	1.28E + 01	2.37E + 01	2.37E + 01
F8	4.27E + 01	2.31E + 01	4.29E + 01	2.71E + 01	3.56E + 01	3.13E + 01	3.13E + 01
F9	4.84E + 01	2.55E + 00	3.72E + 01	5.13E + 01	3.80E + 00	3.87E + 01	3.87E + 01
F10	4.95E + 01	1.15E + 01	5.37E + 01	2.31E + 01	5.03E + 01	4.07E + 01	4.07E + 01
F11	2.82E + 01	2.78E + 01	3.32E + 01	4.49E + 01	3.55E + 01	2.69E + 01	2.69E + 01
F12	4.27E + 01	4.62E + 01	4.77E + 01	4.31E + 01	4.74E + 01	4.35E + 01	4.35E + 01
F13	5.60E + 01	4.75E + 01	4.02E + 01	3.09E + 01	3.72E + 01	3.71E + 01	3.71E + 01
F14	6.20E + 01	5.20E + 01	4.60E + 01	5.33E + 01	3.47E + 01	3.29E + 01	3.29E + 01
F15	5.08E + 01	2.17E + 01	3.60E + 01	4.06E + 01	3.16E + 01	3.10E + 01	3.10E + 01
F16	6.14E + 01	5.76E + 01	4.75E + 01	4.22E + 01	4.77E + 01	5.16E + 01	5.16E + 01
F17	5.89E + 01	4.72E + 01	5.34E + 01	4.01E + 01	5.70E + 01	4.43E + 01	4.43E + 01
F18	4.60E + 01	3.45E + 01	3.61E + 01	5.00E + 01	6.52E + 01	3.86E + 01	3.86E + 01
F19	3.83E + 01	1.64E + 01	4.65E + 01	3.20E + 01	3.99E + 01	2.61E + 01	2.61E + 01
F20	4.21E + 01	5.26E + 01	3.18E + 01	5.48E + 01	3.49E + 01	3.83E + 01	3.83E + 01
F21	5.19E + 01	3.09E + 01	3.86E + 01	5.33E + 01	4.84E + 01	4.37E + 01	4.37E + 01
F22	5.57E + 01	6.48E + 00	4.07E + 01	3.53E + 01	2.52E + 01	4.97E + 01	4.97E + 01
F23	6.07E + 01	6.49E + 01	5.80E + 01	5.22E + 01	6.32E + 01	6.14E + 01	6.14E + 01
F24	6.23E + 01	6.76E + 01	6.65E + 01	5.50E + 01	6.67E + 01	5.90E + 01	5.90E + 01
F25	2.21E + 01	3.98E + 01	4.52E + 01	3.55E + 01	3.66E + 01	4.65E + 01	4.65E + 01
F26	3.01E + 01	2.46E + 01	4.55E + 01	3.89E + 01	4.76E + 01	5.20E + 01	5.20E + 01
F27	7.98E + 01	7.58E + 01	7.22E + 01	6.50E + 01	6.02E + 01	6.83E + 01	6.83E + 01
F28	5.05E + 01	4.04E + 01	4.45E + 01	4.12E + 01	4.95E + 01	4.59E + 01	4.59E + 01
F29	5.85E + 01	3.39E + 01	5.74E + 01	6.19E + 01	5.76E + 01	4.61E + 01	4.61E + 01
F30	4.57E + 01	5.20E + 01	4.54E + 01	4.54E + 01	4.54E + 01	3.53E + 01	3.53E + 01

**Table 5:** Comparison of seven algorithms for optimal value of *Median*.

Function	WOA	HHO	GWO	ALO	AVOA	AGTO	SCAGTO
F1	− 3.97E + 00	− 1.26E + 01	6.12E − 01	− 2.81E + 00	− 2.81E + 00	− 7.31E − 01	− 7.31E − 01
F2	3.22E + 01	2.63E + 01	− 1.06E + 01	9.77E + 00	− 5.29E + 00	− 1.12E + 01	− 1.12E + 01
F3	6.98E + 00	− 4.32E + 00	5.24E − 01	1.88E + 01	− 6.19E + 00	1.31E − 01	1.31E − 01
F4	7.69E + 00	9.13E + 00	4.77E − 01	1.08E + 01	5.39E + 00	− 1.19E + 00	− 1.19E + 00
F5	2.72E + 01	1.53E + 01	− 6.07E − 01	2.24E + 00	− 2.46E + 01	1.29E + 00	1.29E + 00
F6	5.88E + 00	8.78E + 00	4.32E + 00	1.89E + 01	1.72E + 01	7.75E − 01	7.75E − 01
F7	− 1.39E + 00	2.02E + 00	9.22E − 03	3.39E + 00	− 9.04E − 01	− 3.58E − 01	− 3.58E − 01
F8	− 1.96E + 01	3.46E + 00	− 2.96E + 00	− 3.83E + 00	− 1.10E + 01	− 2.06E + 00	− 2.06E + 00
F9	− 5.96E + 00	− 3.73E + 00	− 5.02E + 00	1.53E + 00	1.99E + 00	8.11E − 01	8.11E − 01
F10	− 1.28E + 01	− 9.38E + 01	− 5.97E + 00	9.85E + 01	8.62E + 01	− 3.82E + 00	− 3.82E + 00
F11	− 1.11E + 00	− 1.44E + 01	− 3.36E + 00	3.67E + 00	− 8.60E + 00	− 2.68E − 01	− 2.68E − 01
F12	2.98E + 01	2.73E + 01	5.16E + 00	1.69E + 01	1.34E + 01	− 3.05E + 00	− 3.05E + 00
F13	4.05E + 01	4.08E + 01	1.06E + 01	4.86E + 01	5.12E + 01	− 6.08E − 01	− 6.08E − 01
F14	7.21E + 01	6.74E − 01	− 1.91E + 00	5.09E + 01	− 3.33E + 01	5.43E − 01	5.43E − 01
F15	− 1.72E + 00	2.38E + 01	− 5.49E − 01	− 8.02E + 00	3.04E + 01	− 5.75E − 01	− 5.75E − 01
F16	− 1.86E + 01	− 9.13E + 00	− 8.97E + 00	7.46E + 00	2.18E + 00	− 1.54E + 00	− 1.54E + 00
F17	− 2.48E + 01	− 1.32E + 01	− 3.20E − 01	− 1.73E + 01	− 1.94E + 01	− 4.95E − 01	− 4.95E − 01
F18	− 6.07E + 01	− 4.04E + 01	− 5.18E + 00	− 1.56E + 01	− 2.41E + 01	− 4.68E + 00	− 4.68E + 00
F19	3.76E + 00	1.71E + 01	2.45E − 01	6.76E + 00	2.33E − 01	3.53E + 00	3.53E + 00
F20	2.11E + 01	6.33E + 00	9.93E − 01	8.65E + 00	2.10E + 01	− 4.99E + 00	− 4.99E + 00
F21	1.89E + 01	3.36E + 01	1.04E + 00	1.07E + 01	1.00E + 01	8.20E − 01	8.20E − 01
F22	− 3.35E + 00	− 5.70E + 00	− 2.48E − 01	− 1.97E + 01	− 6.74E + 00	− 4.88E + 00	− 4.88E + 00
F23	− 5.11E + 01	− 3.97E + 01	− 8.14E − 01	− 1.59E + 01	− 5.75E + 01	− 9.83E + 00	− 9.83E + 00
F24	− 5.64E + 01	1.88E + 01	− 1.33E + 01	− 6.20E + 00	− 4.80E + 01	− 9.59E + 00	− 9.59E + 00
F25	9.56E + 00	8.10E + 00	1.25E + 00	1.87E + 01	1.29E + 01	8.04E − 01	8.04E − 01
F26	− 1.12E + 00	6.92E + 00	− 1.15E + 01	− 4.11E + 01	− 1.36E + 01	− 8.14E + 00	− 8.14E + 00
F27	4.57E + 00	8.90E + 00	− 2.90E − 01	2.94E + 00	9.40E + 00	− 2.54E + 00	− 2.54E + 00
F28	− 3.32E + 00	8.06E + 00	− 5.54E − 01	− 7.33E + 00	− 1.08E + 01	− 6.30E − 02	− 6.30E − 02
F29	− 2.30E + 01	2.62E + 01	7.48E + 00	6.28E + 01	− 3.91E + 00	− 6.36E − 01	− 6.36E − 01
F30	− 1.61E + 01	2.34E + 01	5.71E − 02	5.71E − 02	5.71E − 02	1.36E + 00	1.36E + 00

**Table 6:** Comparison of seven algorithms for optimal value of *Max*.

Function	WOA	HHO	GWO	ALO	AVOA	AGTO	SCAGTO
F1	7.55E + 01	7.68E + 01	1.00E + 02	7.67E + 01	7.65E + 01	7.69E + 01	7.69E + 01
F2	9.58E + 01	8.88E + 01	9.51E + 01	9.92E + 01	9.11E + 01	9.68E + 01	9.68E + 01
F3	8.83E + 01	1.45E + 01	7.30E + 01	9.42E + 01	5.22E + 01	7.30E + 01	7.30E + 01
F4	9.56E + 01	8.64E + 01	1.00E + 02	7.57E + 01	1.00E + 02	1.00E + 02	1.00E + 02
F5	8.49E + 01	6.64E + 01	7.17E + 01	8.92E + 01	5.95E + 01	8.26E + 01	8.26E + 01
F6	2.70E + 01	3.14E + 01	8.18E + 01	7.37E + 01	9.94E + 01	7.67E + 01	7.67E + 01
F7	3.40E + 01	6.45E + 00	5.90E + 01	3.72E + 01	4.50E + 01	4.74E + 01	4.74E + 01
F8	8.29E + 01	7.80E + 01	6.17E + 01	6.07E + 01	5.16E + 01	5.95E + 01	5.95E + 01
F9	9.63E + 01	3.68E + 00	7.91E + 01	9.34E + 01	1.07E + 01	6.34E + 01	6.34E + 01
F10	1.00E + 02	-6.34E + 01	8.49E + 01	1.00E + 02	1.00E + 02	8.46E + 01	8.46E + 01
F11	6.46E + 01	4.10E + 01	4.81E + 01	9.57E + 01	6.38E + 01	4.47E + 01	4.47E + 01
F12	6.89E + 01	9.88E + 01	1.00E + 02	9.82E + 01	1.00E + 02	9.74E + 01	9.74E + 01
F13	9.82E + 01	9.52E + 01	9.79E + 01	9.30E + 01	1.00E + 02	9.64E + 01	9.64E + 01
F14	1.00E + 02	1.00E + 02	8.26E + 01	9.96E + 01	4.66E + 01	1.00E + 02	1.00E + 02
F15	9.99E + 01	4.08E + 01	8.90E + 01	8.04E + 01	8.31E + 01	7.70E + 01	7.70E + 01
F16	9.68E + 01	8.65E + 01	9.85E + 01	8.40E + 01	9.28E + 01	1.00E + 02	1.00E + 02
F17	9.28E + 01	6.71E + 01	9.74E + 01	6.63E + 01	9.22E + 01	8.98E + 01	8.98E + 01
F18	6.23E + 01	5.41E + 01	9.56E + 01	7.23E + 01	1.00E + 02	9.05E + 01	9.05E + 01
F19	9.91E + 01	4.97E + 01	9.80E + 01	5.77E + 01	8.44E + 01	9.89E + 01	9.89E + 01
F20	8.95E + 01	9.00E + 01	8.77E + 01	8.97E + 01	9.58E + 01	9.81E + 01	9.81E + 01
F21	9.37E + 01	6.78E + 01	7.82E + 01	8.91E + 01	1.00E + 02	9.59E + 01	9.59E + 01
F22	8.66E + 01	1.08E + 01	8.20E + 01	4.44E + 01	8.35E + 01	9.83E + 01	9.83E + 01
F23	7.65E + 01	8.30E + 01	1.00E + 02	9.54E + 01	6.34E + 01	9.43E + 01	9.43E + 01
F24	1.00E + 02	5.93E + 01	1.00E + 02	8.49E + 01	9.83E + 01	9.48E + 01	9.48E + 01
F25	6.70E + 01	1.00E + 02	1.00E + 02	8.12E + 01	9.94E + 01	1.00E + 02	1.00E + 02
F26	8.47E + 01	4.66E + 01	9.72E + 01	3.74E + 01	6.29E + 01	9.67E + 01	9.67E + 01
F27	1.00E + 02	1.00E + 02	1.00E + 02	9.91E + 01	1.00E + 02	1.00E + 02	1.00E + 02
F28	1.00E + 02	9.56E + 01	1.00E + 02	9.29E + 01	1.00E + 02	1.00E + 02	1.00E + 02
F29	1.00E + 02	6.14E + 01	1.00E + 02	1.00E + 02	9.65E + 01	9.99E + 01	9.99E + 01
F30	7.67E + 01	9.91E + 01	9.97E + 01	9.97E + 01	9.97E + 01	6.21E + 01	6.21E + 01

**Table 7:** Comparison of seven algorithms of running time.

Function	WOA	HHO	GWO	ALO	AVOA	AGTO	SCAGTO
F1	7.37E − 02	1.53E − 01	1.17E − 01	4.45E + 00	1.12E − 01	5.66E − 01	1.98E − 01
F2	4.52E − 01	3.56E − 01	1.24E − 01	5.18E + 00	1.23E − 01	3.95E − 01	4.05E − 01
F3	4.50E − 02	1.25E − 01	8.55E − 02	4.93E + 00	8.45E − 02	2.10E − 01	1.55E − 01
F4	4.55E − 02	1.15E − 01	8.37E − 02	4.75E + 00	8.61E − 02	2.43E − 01	1.58E − 01
F5	6.40E − 02	1.64E − 01	1.02E − 01	4.73E + 00	1.01E − 01	2.33E − 01	1.88E − 01
F6	1.22E − 01	4.95E − 01	3.17E − 01	4.43E + 00	1.63E − 01	3.02E − 01	3.23E − 01
F7	6.36E − 02	1.68E − 01	1.25E − 01	4.64E + 00	1.02E − 01	2.19E − 01	1.95E − 01
F8	6.30E − 02	1.62E − 01	1.01E − 01	4.73E + 00	1.02E − 01	2.31E − 01	1.91E − 01
F9	6.45E − 02	1.81E − 01	1.42E − 01	4.57E + 00	1.05E − 01	2.33E − 01	1.97E − 01
F10	7.65E − 02	2.06E − 01	1.85E − 01	4.52E + 00	1.16E − 01	2.49E − 01	2.18E − 01
F11	5.49E − 02	1.42E − 01	9.25E − 02	4.57E + 00	9.34E − 02	2.29E − 01	1.70E − 01
F12	6.36E − 02	1.61E − 01	1.05E − 01	4.61E + 00	1.07E − 01	2.50E − 01	1.99E − 01
F13	5.63E − 02	1.49E − 01	9.47E − 02	4.55E + 00	9.63E − 02	2.23E − 01	1.77E − 01
F14	7.75E − 02	1.99E − 01	2.19E − 01	4.53E + 00	1.17E − 01	2.54E − 01	2.20E − 01
F15	5.12E − 02	1.45E − 01	9.53E − 02	4.52E + 00	9.33E − 02	2.20E − 01	1.73E − 01
F16	6.05E − 02	1.61E − 01	1.00E − 01	4.49E + 00	1.00E − 01	2.33E − 01	1.88E − 01
F17	9.76E − 02	2.55E − 01	4.15E − 01	4.25E + 00	1.38E − 01	2.81E − 01	2.59E − 01
F18	6.11E − 02	1.63E − 01	1.30E − 01	4.52E + 00	9.96E − 02	2.31E − 01	1.85E − 01
F19	3.85E − 01	1.22E + 00	4.17E − 01	4.49E + 00	3.99E − 01	6.06E − 01	7.75E − 01
F20	1.24E − 01	3.47E − 01	2.88E − 01	4.27E + 00	1.45E − 01	2.92E − 01	2.79E − 01
F21	1.25E − 01	3.83E − 01	3.10E − 01	4.23E + 00	1.63E − 01	3.05E − 01	3.11E − 01
F22	1.45E − 01	4.64E − 01	3.00E − 01	4.24E + 00	1.80E − 01	3.32E − 01	3.43E − 01
F23	1.53E − 01	5.12E − 01	2.60E − 01	4.27E + 00	1.87E − 01	3.40E − 01	3.54E − 01
F24	1.71E − 01	6.15E − 01	2.45E − 01	4.23E + 00	2.08E − 01	3.67E − 01	4.03E − 01
F25	1.29E − 01	4.13E − 01	3.18E − 01	4.25E + 00	1.67E − 01	3.11E − 01	3.18E − 01
F26	1.82E − 01	6.64E − 01	2.48E − 01	4.24E + 00	2.28E − 01	3.76E − 01	4.18E − 01
F27	2.06E − 01	7.29E − 01	2.63E − 01	4.31E + 00	2.44E − 01	4.03E − 01	4.69E − 01
F28	1.63E − 01	5.74E − 01	2.57E − 01	4.22E + 00	1.97E − 01	3.61E − 01	3.85E − 01
F29	1.46E − 01	5.53E − 01	2.38E − 01	4.22E + 00	1.84E − 01	3.34E − 01	3.63E − 01
F30	4.53E − 01	1.16E + 00	4.59E − 01	4.41E + 00	4.45E − 01	6.56E − 01	8.69E − 01

From Tables 2, 3, 4, 5, 6, and 7, it can be observed that the SCAGTO algorithm presented in this paper fails to achieve the theoretical optimal value only under the functions F1-F8, and achieves the theoretical optimal value in the remaining 22 test functions in various dimensions, demonstrating a strong ability to identify the optimal value. Regarding the 30 test functions, the SCAGTO algorithm exhibits a disadvantage only in the robustness of F1–F5. The standard deviation of the optimization results in all dimensions of the remaining test functions is 0, indicating the strong stability of the SCAGTO algorithm. Analyzed in terms of dimensionality, as the dimensionality of the test functions increases, the optimization ability and robustness of the algorithms AGTO, WOA, GWA, and ALO show an overall decreasing trend. In terms of the standard deviation, the value of the SCAGTO algorithm is generally smaller than that of the AGTO algorithm, also indicating that the introduction of the positive cosine strategy and Cauchy's variant has enhanced the robustness of the AGTO algorithm.

Typically, the time complexity of an algorithm can be assessed based on the time it takes to run. The time complexity reflects the superiority of the algorithm. As the complexity of the algorithm increases, it indicates that the algorithm operates less efficiently. With 30 test functions, the complexity of solving the problem rises as the dimensionality increases, resulting in a longer overall execution time. In terms of algorithm comparison, the AVOA algorithm takes the shortest time overall, and the SCAGTO algorithm takes longer compared to the improved AGTO, WOA, GWA, and ALO algorithms. This indicates that the introduction of the improved strategy does not enhance the complexity of the AGTO and reduces the execution efficiency. The SCAGTO algorithm takes longer than the AGTO algorithm because it combines both global search and local exploitation. However, for some functions with different dimensions, the AGTO algorithm exhibits a time consumption comparable to the SCAGTO algorithm. This is primarily attributed to the introduction of several improvement strategies, which expands the range of optimisation search of the SCAGTO algorithm, requiring more time and consequently increasing the time consumption. To summarize, the effectiveness of the improvement strategies proposed in this paper is analyzed and verified based on the conclusions in Fig. 5 and Tables 2, 3, 4, 5, 6, and 7.

### Applications to engineering optimisation problems

Swarm intelligence optimization algorithms offer several advantages in engineering applications, including efficiency in finding optimal solutions for large-scale problems, adaptability through automatic search strategy adjustments, robustness in handling complex engineering issues, suitability for distributed computing, and ease of implementation. These algorithms find applications in various fields such as logistics and supply chain management, electrical engineering, mechanical engineering, communication engineering, chemical engineering, transportation, financial engineering, image processing, and computer vision, as well as bioinformatics. The ability to optimize processes, designs, and scheduling, along with handling tasks like wireless network planning, signal processing, and risk management, makes them valuable in these domains. This article applies the proposed improved gorilla optimization algorithm to the pressure vessel design optimization (PVD) in engineering design and the classic problem of optimized design problem for welded beams, and compares it with other algorithms, in order to solve practical engineering problems.

#### Pressure vessel design optimisation (PVD)

To verify the superiority of SCAGTO in real engineering, the pressure vessel design optimisation problem is selected and compared by validation with six other algorithms. The pressure vessel design problem is a classical engineering optimisation design problem that aims to reduce the manufacturing cost by reducing the consumables of the pressure vessel^53^. The pressure vessel is capped at both ends by a lid and the head end consists of a hemispherical lid. The design problem consists of four main variables: i.e. the cross-sectional length (*L*) of the cylindrical portion that is not the head, the inner wall diameter (R), the wall thickness (*T*_*s*_) and the wall thickness of the head (*T*_*h*_)^54^. The main objective of the pressure vessel design problem is to minimize the manufacturing cost while ensuring the functionality of the pressure vessel by choosing four variables: shell thickness (*T*_*s*_), head thickness (*T*_*h*_), inner wall radius (*R*), and cylindrical section length (*L*). The mathematical model is represented as follows:

(1) Variable design:

26 $$ x = [x_{1} ,x_{2} ,x_{3} ,x_{4} ] = [T_{s} ,T_{h} ,R,L] $$

(2) Objective function:

27 $$ \min f(x) = 0.6224x_{1} x_{3} x_{4} + 1.77x_{2} x_{3}^{2} + 3.1661x_{1}^{2} x_{4} + 19.84x_{1}^{2} x_{3} $$

(3) Constraints:

28 $$ g_{1} (x) = - x_{1} + 0.0193x_{3} \le 0 $$

29 $$ g_{2} (x) = - x_{2} + 0.00954x_{3} \le 0 $$

30 $$ g_{3} (x) = - \pi x_{3}^{2} x_{4} - \frac{3}{4}\pi x_{3}^{3} + 1296000 \le 0 $$

31 $$ g_{4} (x) = x_{4} - 240 \le 0 $$

(4) Boundary constraints:

$$0 \le x_{1} \le 99, \, 0 \le x_{2} \le 99$$, $$10 \le x_{3} \le 220, \, 10 \le x_{4} \le 200$$.

**Figure 6:**
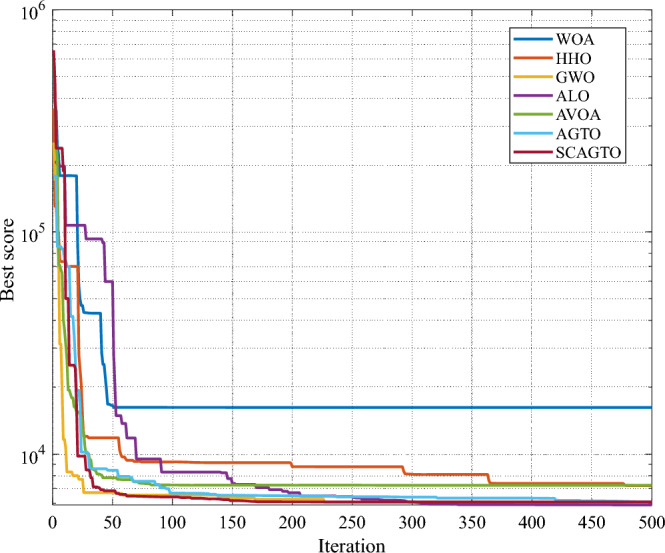
Iterative process of optimal solutions for pressure vessel design optimization.

The iterative process of finding the optimal solution of the seven algorithms is shown in Fig. 6. The previous simulation experiments have verified that scagto algorithm has good performance in unconstrained test functions, and the pressure vessel design problem as a classical constraint problem can prove that scagto algorithm also has advantages in solving constrained problems. In the experiment, Table 8 lists the experimental results of scagto and other six algorithms in solving pressure vessel design problems.

**Table 8:** Performance comparison of pressure vessel design algorithms.

Algorithms	*T*_*s*_	*T*_*h*_	*R*	*L*	*f*(*x*)
WOA	1.33	6.62	45.33	140.25	11,069.36
HHO	1.37	8.23	52.35	80.72	6871.86
GWO	1.3245	7.2	42.09	176.75	6086.84
ALO	1.35	7.35	41.89	179.21	6538.94
AVOA	1.32	6.87	42.09	176.65	6066.09
AGTO	1.25	6.57	57.99	157.74	6233.64
SCAGTO	1.15	6.03	40.32	179.21	6059.71

From Table 8, the SCAGTO algorithm obtains the optimal solution 6059.71 at *T*_*s*_ = 1.15, *T*_*h*_ = 6.03, *R* = 40.32, *L* = 179.21, which are all significantly better than six algorithms such as whale optimisation algorithm (WOA), harris hawk algorithm (HHO), grey wolf optimisation algorithm (GWO), ALO algorithm and AVOA algorithm. It is shown that the SCAGTO algorithm is able to solve the pressure vessel design problem with the lowest cost, which is suitable for solving this type of constrained engineering design problem, and has more superior performance compared with other algorithms. The proposed improved the SCAGTO algorithm can circumvent the shortcomings of the basic AGTO algorithm in the constrained optimisation problem, and better solve the constrained optimisation problem in pressure vessel design. In summary, SCAGTO has good performance on both unconstrained and constrained problems, and can be applied to solving mathematical problems as well as real-world problems.

#### Optimized design problem for welded beams

The objective of the welded beam design problem is to reduce the manufacturing cost of the welded beam while ensuring safety performance, and the four variables to be optimized for this problem are the weld seam width *h*, the length of the connecting beam *L*_*s*_, the height of the connecting beam *t*_*s*_, and the thickness of the connecting beam *b*^55^.

The objective function is

32 $$ f_{\min } = 1.10471h^{2} L_{s} + 0.04811t_{s} b\left( {14 + L_{s} } \right) $$

The internal constraint parameters include shear stress *τ*, beam bending stress *σ*, beam deflection *β*, buckling load *W*, internal pressure *E* on the welded beam, external pressure *G* on the welded beam, external length of the beam *L*, partial load amount *M*, and axial surface force *U*. The internal constraint parameters are as follows: *τ*_*max*_ is 13,600 N, *σ*_*max*_ is 30,000 N, *β*_*max*_ is 0.25 m, *W* is 6,000 N, *G* is 1.2 × 10^7^ N. The relationship between partial parameters and variables is^56^: *τ*_*max*_ is 14 cm, and E is 3 × 10^7^ N. The relationship between partial parameters and variables is Ref.^56^: *τ*_*max*_ is 1.2 × 10^7^ N. *L* is 14 cm, and *E* is 3 × 10^7^ N. Some of the parameters are related to the variables as Ref.^56^.

33 $$ \tau ^{\prime} = \frac{P}{{2hL_{s} }},\tau ^{\prime\prime} = MUJ $$

34 $$ M = W\left( {L + \frac{{L_{s} }}{2}} \right) $$

35 $$ J = 2\sqrt 2 hL_{s} \left[ {\frac{{L_{s}^{2} }}{12} + \left( {\frac{{h + t_{s} }}{2}} \right)^{{^{2} }} } \right] $$

36 $$ U = \sqrt {\frac{{L_{s}^{2} }}{4} + \left( {\frac{{h + t_{s} }}{2}} \right)^{{^{{^{2} }} }} } $$

The constraints are:

37 $$ \sqrt {(\tau ^{\prime})^{2} + \frac{{\tau ^{\prime}\tau ^{\prime\prime}L_{s} }}{R} + (\tau ^{\prime\prime})^{2} } \le 0 $$

38 $$ \frac{6WL}{{t_{s}^{2} b}} - \sigma_{\max } \le 0 $$

39 $$ h - b \le 0 $$

40 $$ 1.10471h^{2} L_{s} + 0.04811t_{s} b(14 + L_{s} ) - 5 \le 0 $$

41 $$ 0.125 - \frac{h}{cm} \le 0 $$

42 $$ \frac{{4WL^{3} }}{{Et_{s}^{2} b}} - \delta_{\max } \le 0 $$

43 $$ W - \frac{{4.013Et_{s} b^{3} }}{{6L^{2} }}\left( {1 - \frac{{t_{s} }}{2L}\sqrt{\frac{E}{4G}}  } \right) \le 0 $$

Boundary constraints:

44 $$ 0.1 \le h, \, L_{s} \le 2, \, 0.1 \le t_{s} , \, b \le 10 $$

**Figure 7:**
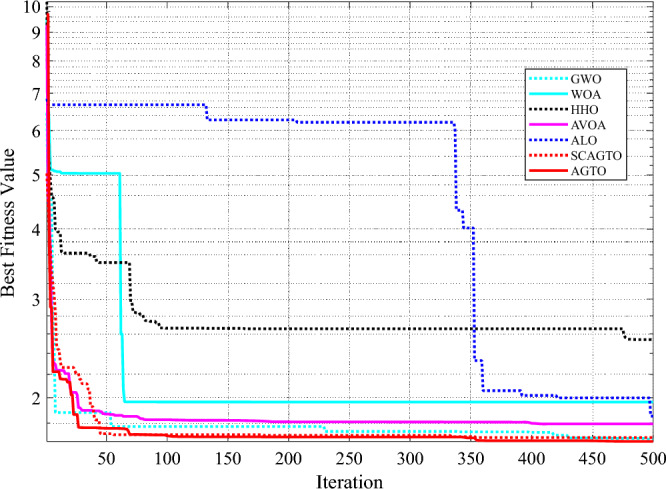
Iterative process of the optimal solution for optimized design of welded beams.

The SCAGTO algorithm is compared with six algorithms, including Whale Optimisation Algorithm (WOA), Harris Hawk Algorithm (HHO), Grey Wolf Optimisation Algorithm (GWO), ALO and AVOA. Each algorithm is run independently for 50 times, and the average of the results is taken as the optimal solution. The iterative process of the seven algorithms to find the optimal solution is shown in Fig. 7. The simulation experiments in the previous section have verified that the SCAGTO algorithm has a good performance on the unconstrained test function, and the welded beam design problem as a classical constrained problem can prove that the SCAGTO algorithm is also superior in solving the constrained problem. In the experiments Table 9 lists the experimental results of SCAGTO and other six algorithms in solving the welded beam design problem.

**Table 9:** Comparison of optimized design algorithms for welded beams.

Algorithms	*h/cm*	*L*_*s*_/cm	*t*_*s*_/cm	*b/cm*	*f*_min_
WOA	0.125	5.8389	8.6056	0.2268	1.9641
HHO	0.43846	1.9484	6.0635	0.4569	2.5397
GWO	0.1892	3.5305	9.1878	0.1992	1.683
ALO	0.1465	5.1312	8.9851	0.2093	1.8531
AVOA	0.1319	5.2554	9.1924	0.1988	1.7942
AGTO	0.1988	3.3374	9.1926	0.1994	1.6702
SCAGTO	0.198	3.3531	9.1821	0.1988	1.6711

It can be seen that the SCAGTO algorithm obtains the largest weld seam width, the smallest beam length, the moderate beam width and beam thickness, and the smallest minimum cost, which indicates that SCAGTO is more advantageous in solving the welded beam design problem.

By solving the above two engineering problems, it is further verified that the SCAGTO algorithm can find a better solution in the actual engineering problems, which has a high value of engineering applications.

## Conclusions

In this paper, an improved artificial gorilla troop optimization algorithm (SCAGTO) that combines the sine–cosine algorithm and the Cauchy variant is proposed. To overcome the problem of gorilla stasis in the later stages of the search caused by insufficient population diversity, a refractive backward learning mechanism is introduced in the population initialization. Additionally, a positive cosine mechanism, a nonlinear decreasing search factor, and a weight factor are added to the gorilla discoverer's position to better address the shortcomings of the AGTO algorithm in balancing global and local optimization. Cauchy's variation in the gorilla follower is utilized to mutate the current optimal individual, thereby expanding the feeding range of the gorillas and consequently enhancing the global optimization accuracy and speed of the AGTO algorithm. The SCAGTO algorithm is examined using 30 classical test functions and compared in terms of convergence speed and accuracy tests with other algorithms and the latest gorilla algorithm improvement strategies. The results indicate that the Gorilla search algorithm, which incorporates positive cosine and Cauchy variants, exhibits enhanced performance in global optimization and local exploitation, thereby validating the effectiveness and reliability of the improvement strategy.

The simulation experiments employ higher-dimensional benchmark functions to test the optimization performance of the improved algorithm, while other improved SCAGTO algorithms and popular algorithms are selected for comparison. The experimental results reveal that SCAGTO demonstrates better convergence speed and accuracy for different types of test functions, showcasing a certain degree of competitiveness and stability. Finally, we verify the practical problem-solving capabilities of the improved algorithm through two classical engineering design problems, namely, pressure vessel optimization and welded beam design. The results show that SCAGTO can be applied to solve practical problems and holds promise for engineering applications. The SCAGTO algorithm exhibits certain advantages in optimizing the pressure vessel design problem and the welded beam design problem, confirming the superior optimization capabilities and engineering practicality of the SCAGTO algorithm. However, the introduction of SCAGTO inevitably leads to an increase in running time. Therefore, the future work direction is to make continuous improvements on the existing basis, ensuring optimization performance while reducing running time overhead and enhancing computational efficiency. Secondly, SCAGTO can be applied to multi-objective optimization and further explored in conjunction with practical problems. The follow-up work considers the in-depth application of SCAGTO to other mechanical process optimization designs for the resolution of practical problems.

## Data Availability

The data that support the findings of this study are available from the corresponding author upon request. There are no restrictions on data availability.
